# Increased risk of chronic kidney disease and mortality in a cohort of people diagnosed with metabolic dysfunction associated steatotic liver disease with hepatic fibrosis

**DOI:** 10.1371/journal.pone.0299507

**Published:** 2024-04-16

**Authors:** Marc Gurun, Paul Brennan, Sava Handjiev, Aseil Khatib, Damien Leith, John F. Dillon, Christopher J. Byrne

**Affiliations:** 1 Molecular and Clinical Medicine, School of Medicine, University of Dundee, Ninewells Hospital and Medical School, Dundee, United Kingdom; 2 Department of Gastroenterology, NHS Tayside, Ninewells Hospital and Medical School, Dundee, Scotland; 3 Department of Biochemical Medicine, NHS Tayside, Ninewells Hospital and Medical School, Dundee, Scotland; 4 Population Health and Genomics, School of Medicine, University of Dundee, Ninewells Hospital and Medical School, Dundee, United Kingdom; 5 Directorate of Public Health, NHS Tayside, Kings Cross Hospital, Dundee, United Kingdom; Universita degli Studi della Campania Luigi Vanvitelli Scuola di Medicina e Chirurgia, ITALY

## Abstract

**Background and aims:**

Metabolic dysfunction associated steatotic liver disease (MASLD) increases the risk of incident chronic kidney disease (CKD). However, the relative risk of CKD associated with increasing hepatic fibrosis, and consequent mortality risk, remains underexplored in real-world cohorts. In this study, we sought to establish whether hepatic fibrosis is associated with increased CKD risk and explore differences in mortality risk in a cohort of people living with MASLD, contingent on liver fibrosis and CKD status.

**Methods:**

This was an observational study of people who underwent routine liver function testing in Tayside, Scotland. MASLD was defined as: elevated ALT (>30 U/L) or GGT (>73 U/L); presence of diabetes, and/or hypertension, and/or obesity; weekly alcohol consumption <14 units (112g (+/-8g) alcohol); and negative screen for other aetiologies. Data was collected from digital health records. We used log-binomial models to quantify the risk of CKD among those with and without fibrosis, and Cox regression models to estimate differences in mortality risk dependent on fibrosis and CKD.

**Results:**

In our cohort (n = 2,046), 1,448 (70.8%) people had MASLD without fibrosis and 598 (29.2%) with fibrosis; 161 (11.1%) and 117 (19.6%) respectively also had CKD. After excluding individuals with structural, autoimmune, or malignant CKD (n = 22), liver fibrosis (n = 593; 18.9% with CKD) was associated with increased CKD risk (*a*RR = 1.31, 1.04–1.64, p = 0.021). Increased mortality risk was observed for those with liver fibrosis (*a*HR = 2.30, 1.49–3.56, p = <0.001) and was higher again among people with both fibrosis and CKD (*a*HR = 5.07, 3.07–8.39, p = <0.014).

**Conclusions:**

Liver fibrosis was an independent risk factor for CKD in this cohort of people living with MASLD. Furthermore, those with MASLD with liver fibrosis had higher risk for mortality and this risk was further elevated among those with co-morbid CKD. Given the increased risk of CKD, and consequent mortality risk, among people living with MASLD fibrosis, renal function screening should be considered within liver health surveillance programmes and guidelines.

## Introduction

Metabolic dysfunction associated steatotic liver disease (MASLD) and chronic kidney disease (CKD) are widespread chronic conditions, with estimated prevalence of 32.4% (29.9–34.9) and 13.4% (11.7–15.1) respectively [1, 2]. Beyond morbidity, both are leading determinants of premature mortality from causes such as cardiovascular disease (CVD) [2, 3]. MASLD is closely linked with the metabolic syndrome (MetS), which encompasses hypertension, obesity, type 2 diabetes mellitus, hyperlipidaemia, and CVD [4, 5]. MASLD has become a major public health concern, and to inform and enhance healthcare provision for those at risk, there is a need to further understand associated comorbidities such as, in addition to conditions of the MetS, extrahepatic malignancies, hypothyroidism, renal calculi, biliary colic, depression, and CKD [6–9].

There is growing evidence of a relationship between MASLD and CKD. Studies have indicated MASLD diagnosis, and possibly severity, is associated with heightened risk of CKD acquisition [10–17]. There is uncertainty, however, about whether this effect is driven by the full MASLD spectrum (encompassing simple steatosis, metabolic dysfunction associated steatohepatitis (MASH), with significant and advanced fibrosis, and cirrhosis), components of the MetS, or by the more advanced hepatic consequences of MASLD, namely progressive fibrosis. There are few studies which examine this, particularly adjusted for relevant confounders, in real-world MASLD-only cohorts with validated liver fibrosis markers, as reliable diagnostic methods such as transient elastography and non-invasive tests (NIT) remain somewhat inaccessible [18, 19]. Examining the relationship between hepatic fibrosis and CKD in the MASLD context, however, is important to facilitate prioritisation of interventions for those at high CKD risk among the large numbers of people living with MASLD. Furthermore, should such a relationship exist, this would enhance our understanding of pathophysiological mechanisms linking MASLD and CKD, which remain debated. Proposed mechanisms include renin-angiotensin dysfunction, fructose metabolism, gastrointestinal dysbiosis, oxidative stress and cellular senescence [19, 20].

In this work, we focus on the relationship between MASLD, liver fibrosis, and CKD to examine the interdependence between MASLD fibrosis and CKD, and any potential synergistic effect. We undertook an observational cohort study among people diagnosed with MASLD and aimed to establish whether liver fibrosis is associated with increased CKD risk, independent of other important confounders. Previous studies have compared MASLD and non-MASLD cohorts, however here we compare people with MASLD and liver fibrosis to those with MASLD but without fibrosis. In isolating fibrosis as a causative factor, we aimed to assess whether the association between MASLD and CKD previously identified in studies comparing MASLD and non-MASLD participants may be associated with fibrogenesis, and consequently provide some insight into its aetiology. We also aimed to compare differences in mortality stratified by MASLD fibrosis and CKD status, to understand any potential differences in subsequent risk in this co-morbid context.

## Methods

### Study design

This was an observational cohort study of people who underwent National Health Service (NHS) Tayside’s intelligent liver function testing (iLFT) cascade from 09/03/2016 to 31/08/2022. Data was accessed from 02/02/2023 to 01/04/2023. iLFT is an automated diagnostic algorithm that assists primary care physicians in the management and referral of people who present with abnormal liver function tests (LFT) [21]. iLFT applies sequential aetiological investigations consequent to abnormal LFTs that includes immunological markers, viral serology, iron studies and non-invasive fibrosis tests [NIT]. This generates preliminary diagnoses and management plans for the individual. The study population consists of people for whom iLFT generated a diagnosis of MASLD. Participants were observed from the date of their iLFT to the end of the study observation period: 01/04/2023.

### Setting

Tayside is a geographical region in the east of Scotland with an estimated population of 417,000, split between the region’s two urban centres and surrounding rural areas. Healthcare–free at the point of need–is delivered through the publicly-funded NHS. There is socioeconomic diversity within the region, and consequently varying health outcomes across socio-economic indices; like the rest of Scotland, obesity and CVD are common [22].

### Participants

This study included adults (≥18 years) who underwent iLFT screening during the observation period. The authors, who held relevant Caldicott Guardian approval, had access to information that could identify participants during data collation. Once data was collated, and prior to analysis, participants were de-identified and allocated random ID numbers. Participants were stratified into having either MASLD with or without fibrosis for the primary analysis, based on available NITs.

### Variables

The following data was collected: demographic factors (age, sex, Body Mass Index [BMI], smoking history, deprivation status); serum biochemical results (alanine transaminase [ALT], albumin, aspartate aminotransferase [AST], gamma-glutamyltransferase [GGT], platelets, creatinine, estimated glomerular filtration rate [eGFR]); urinary albumin-to-creatinine rations (uACR) results; NITs (transient elastography [TE], enhanced liver fibrosis [ELF] test, MASLD fibrosis score [NFS], fibrosis-4 index [FIB-4]); comorbidities (hypertension, diabetes, cardiovascular disease); mortality status; prescribed medications and, if diabetic, the HbA1c result closest to iLFT date.

### Variable definitions

**Fig 1:**
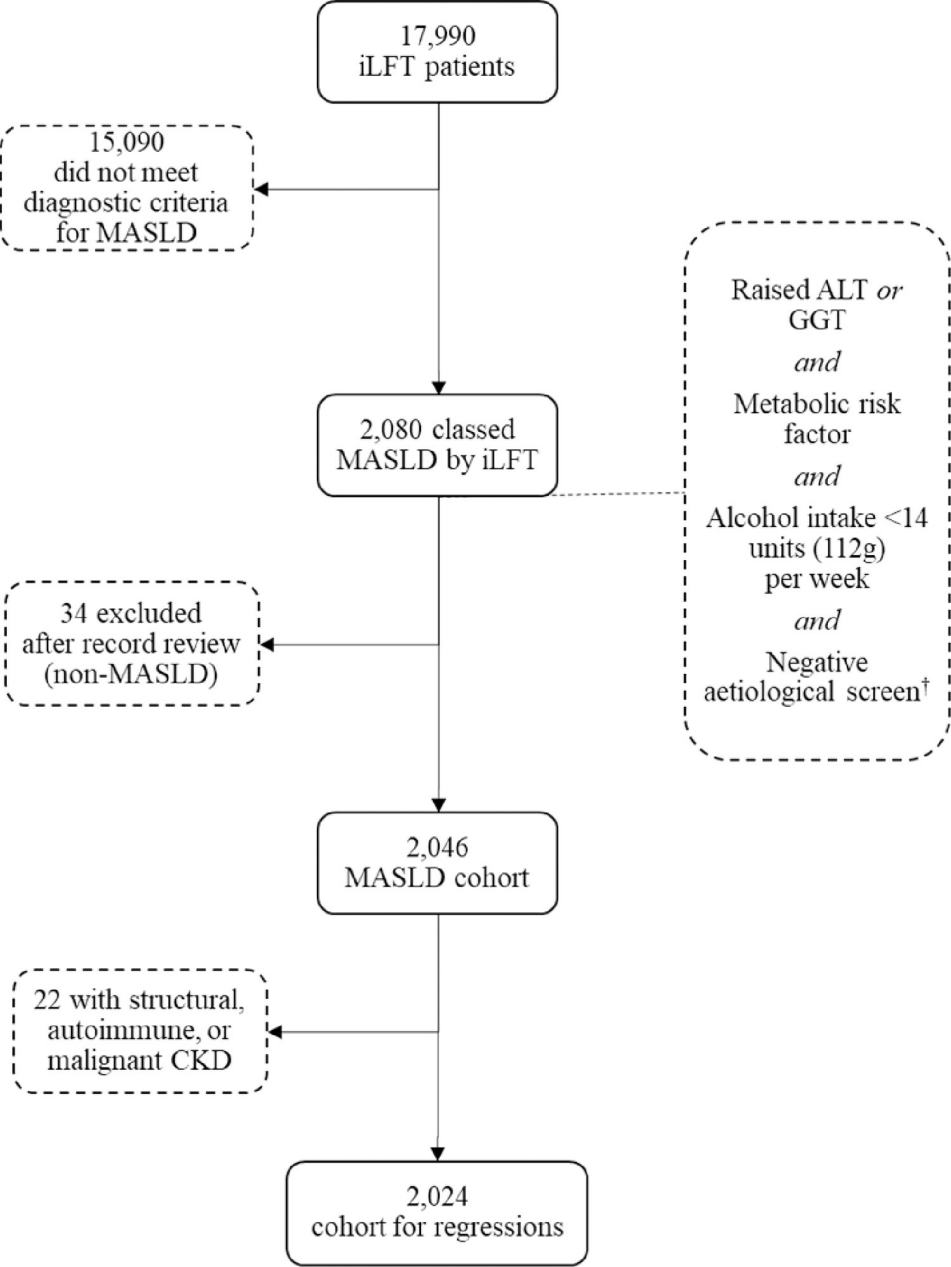
Participant flow diagram.

The diagnostic criteria for MASLD used in iLFT [21] are:

1. Elevated ALT (>30 U/L) or GGT (>73 U/L) (evidencing steatosis).
2. Presence of a least one of the following metabolic factors: diabetes, hypertension, obesity.
3. Weekly alcohol consumption <14 units (112g (+/-8g) alcohol per week).
4. Negative screen for other aetiologies (Fig 1).

This, although simplified, aligns well with recent MASLD consensus guidelines [23]. Case notes were reviewed and people with non-MASLD liver disease aetiology were excluded. Fibrosis (of any degree) was defined as liver stiffness measurement (LSM) ≥8kPa on TE, ELF score ≥9.8, NFS ≥0.675, FIB-4 ≥2.67 or ALT:AST ratio >1. Absence of fibrosis was defined as TE LSM <8kPa, ELF score <9.8, NFS <-1.455, FIB-4 <1.45 or ALT:AST ratio <1. Indeterminate NFS and FIB-4 was a result between -1.455 to 0.675 and 1.45 to 2.67 respectively. These tests were applied hierarchically to define fibrosis status, where TE was used first if available; if unavailable, ELF was used, and so on, through to AST:ALT ratio. CKD status was established using the Kidney Disease Improving Global Outcomes diagnostic criteria, with a requirement of two or more reduced eGFR (calculated using the chronic kidney disease epidemiology collaboration equation) or elevated uACR results greater than three months apart [24]. Diabetes was defined as the presence of an elevated HbA1c result or documented diagnosis. CVD was defined as a history of ischaemic heart disease, cerebrovascular disease, or peripheral vascular disease. We defined renal-impairing medications as those identified in the British National Formulary, [25] as well as medications that are known to reduce GFR, namely angiotensin-converting enzyme (ACE) inhibitors, angiotensin receptor blockers (ARBs), and diuretics (Table 1) [26, 27].

**Table 1:** Total cohort demographic, renal, and prescribing parameters (n = 2,046).

Characteristic	Measure
Sex	
Male	1,144 (55.9%)
Female	902 (44.1%)
Age	
Median (IQR)	61 (52–70)
Deprivation quintile^†^	
1 (least socio-economic resource)	482 (23.6%)
2	418 (20.4%)
3	552 (27.0%)
4	219 (10.7%)
5 (most socio-economic resource)	375 (18.3%)
Body Mass Index	
≤30	817 (39.9%)
>30	1,229 (60.1%)
Ever smoked	
No	1,045 (51.1%)
Yes	1,001 (48.9%)
Chronic Kidney Disease^‡^	
No	1,768 (86.4%)
Yes	278 (13.6%)
Repeat prescriptions	
Nephrotoxic medications^¶^	
No	1,945 (95.0%)
Yes	101 (5.0%)
Nephrotoxic medications and/or ACE inhibitors and/or ARBs and/or diuretics^§^	
No	1,008 (49.3%)
Yes	1,038 (50.7%)
Beta blockers^||^	
No	1600 (78.2%)
Yes	446 (21.8%)

**Abbreviations:** IQR, inter-quartile range; ACE, angiotensin-converting-enzyme; ARB, angiotensin receptor blockers.

^†^Derived using postcode of participants’ general practitioners cross-referenced against the Scottish Index of Multiple Deprivation.

^‡^Defined as two consecutive estimated glomerular filtration rate values <60 mL/min/1.73m2 and/or two consecutive urine albumin to creatinine ratio values of ≥3mg/mmol, within one year of intelligent liver function testing (six months prior/post).

^¶^Aceclofenac, aciclovir, adefovir, amikacin, amphotericin B, bacitracin, capreomycin, carboplatin, cefaclor, cefadroxil, cefalexin, cefazolin, cefepime, cefixime, cefotaxime, cefoxitin, cefradine, ceftaroline, ceftazidime, ceftobiprole, ceftolozane, ceftriaxone, cefuroxime, celecoxib, ciclosporin, cidofovir, cisplatin, colistemethate, dexibuprofen, dexketoprofen, diclofenac, etodolac, etoricoxib, flurbiprofen, foscarnet, ganciclovir, gentamicin, ibuprofen, ifosfamide, indometacin, ketoprofen, ketorolac, mefenamic acid, meloxicam, methotrexate, nabumetone, naproxen, neomycin, oxaliplatin, parecoxib, pemetrexed, penicillamine, pentamidine, phenazone, piroxicam, polymyxin b, streptomycin, streptozocin, sulindac, tacrolimus, telavancin, tenofovir disoproxil, tenoxicam, tioprofenic acid, tobramycin, tolfenamic acid, trimethoprim, valaciclovir, valganciclovir, vancomycin, zidovudine, zoledronate.

^§^Enalapril, lisinopril, perindopril and ramipril; candesartan, irbesartan, losartan, valsartan and olmesartan; indapamide and bendroflumethiazide.

^||^Atenolol, bisoprolol, carvedilol, propranolol, metoprolol.

### Data source

Data was collected from digital medical records and NHS Tayside’s laboratory reporting service.

### Ethics

As this was not an interventional study, but rather a retrospective evaluation using routine health data held in patient records, NHS research ethics review was not required. Prior to data collection, approval for data access was obtained from NHS Tayside’s Caldicott Guardian (reference: IGTCAL11585). This is a regulated approval process that ensures safe and appropriate use of identifiable health data, in lieu of explicit consent from patients, which was not feasible for this population-level retrospective work [28, 29].

### Study size

Over the observation period, iLFT cascaded for 17,990 people, of whom 2,080 were attributed a working diagnosis of MASLD. Thirty-four individuals with a non-MASLD liver diagnosis on review of their medical records were excluded, giving a study cohort of n = 2,046. Further, 22 individuals were excluded from the regression analyses due to having structural, autoimmune, or malignant CKD, meaning the sample size for statistical analyses was n = 2,024.

### Statistical methods

Descriptive statistics were used to derive counts and proportions. We estimated risk ratios using log-binomial regression. We estimated survival time using Kaplan-Meier methods and hazard ratios using Cox regression. Kaplan-Meier survival patterns were compared using the Logrank test. Participants with CKD of structural, malignant, or autoimmune aetiology were excluded from regression and survival analyses. The following independent variables were included in the adjusted Cox regression: MASLD fibrosis status, age (split on median), sex, BMI, hypertension, diabetes, CVD, smoking history, and repeat prescription of renal-impairing medications. All variables were binary and were force entered as they were theoretically rationalised. Multicollinearity was assessed, with a variance inflation factor threshold of two. For the survival analyses, attained age was used for the time scale rather than time on study [30, 31]. Age at entry was on date of screening and age at exit was at date of death or the censor date (01/04/2023), whichever occurred first. Thus, the models are implicitly adjusted for age, and it was not entered as an independent variable. The terminating event was death. The following variables were included in the adjusted survival analysis: MASLD fibrosis status, sex, diabetes, and CVD. The proportional hazards assumption was inspected using the -estat phtest- and -stphplot- functions. All statistical analysis was performed using Stata 17and *p* values of ≤0.05 were assumed to infer statistical significance.

## Results

During the observation period, 17,990 people underwent iLFT screening, of whom 2,080 (11.6%) were assigned an iLFT diagnosis of MASLD. Following record review, 34 (0.2%) individuals with non-MASLD liver disease aetiologies were excluded, leaving a cohort of 2,046 (11.4%) participants with MASLD (Fig 1). Of those, 70.8% (n = 1,448) had MASLD without fibrosis, while 29.2% (n = 598) had MASLD with fibrosis.

Demographic, renal, and prescribing parameters are outlined in Table 1. Most participants (n = 1,144; 55.9%) were male and 900 (44.0%) lived in areas of least socio-economic resource. Median age and BMI were 61 (52–70) and 31 (28–35) respectively. Five percent (n = 101) had repeat prescription of nephrotoxic medication, while 21.8% were prescribed beta blockers. A history of ever smoking was noted among 48.9% (n = 1,001) of participants.

**Fig 2:**
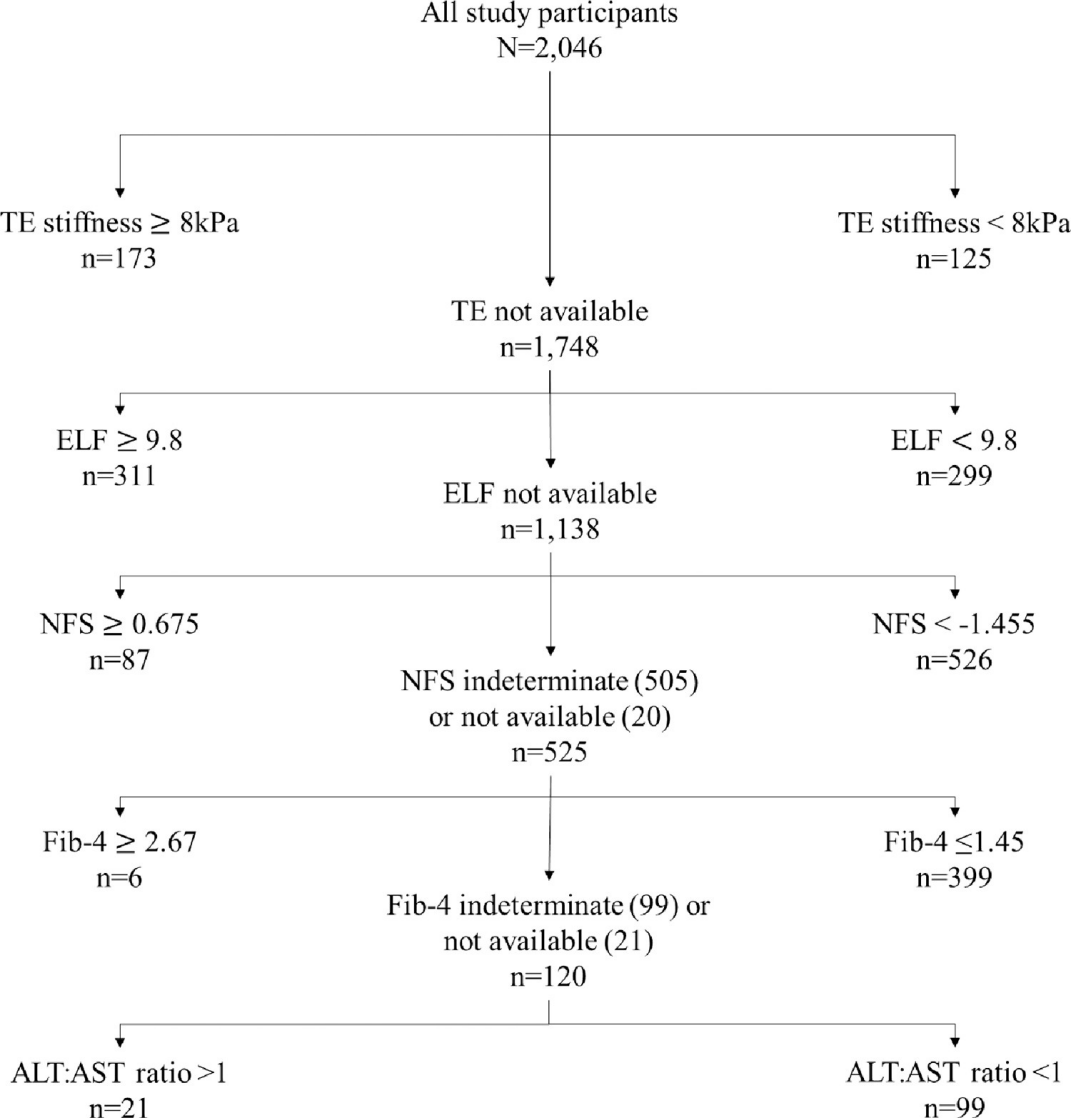
Flowchart showing the classification algorithm used to identify participants as either MASLD with fibrosis (left) or MASLD without fibrosis (right).

The overall prevalence of CKD was 13.6% (n = 278), diabetes 71.4% (n = 1,461), CVD 24.0% (n = 490), and hypertension 54.6% (n = 1,117). Of those with CKD, 40.7% (n = 113) were diagnosed by reduced eGFR, 43.2% (n = 120) with elevated ACR, while 16.2% (n = 45) had evidence of both. CKD grading classified 53.8% (n = 150) to be at moderately increased risk, 28.2% (n = 78) at high risk and 18.0% (n = 50) at very high risk of having CKD. NITs used to determine fibrosis status are outlined in Fig 2, and median NIT values stratified by fibrosis status are outlined in Table 2.

**Table 2:** Fibrosis biomarker results for the cohort (n = 2,046).

Non-invasive Test	Median (IQR)
TE LSM kPa (n = 298)	
Fibrosis present (n = 173)	11.4 (9.5 to 16.4)
Fibrosis absent (n = 125)	6.0 (5.0 to 6.8)
ELF (n = 610)	
Fibrosis present (n = 311)	10.7 (10.2 to 11.6)
Fibrosis absent (n = 299)	9.2 (8.8 to 9.5)
NFS (n = 613)	
Fibrosis present (n = 87)	1.258 (0.879 to 0.921)
Fibrosis absent (n = 526)	-2.097 (-2.686 to -1.728)
Fib-4 (n = 405)	
Fibrosis present (n = 6)	4.62 (2.99 to 11.39)
Fibrosis absent (n = 399)	0.99 (0.81 to 1.18)
AST:ALT ratio (n = 120)	
Fibrosis present (n = 21)	1.11 (1.05 to 1.25)
Fibrosis absent (n = 99)	0.69 (0.60 to 0.86)

**Abbreviations:** TE LSM, Transient elastography liver stiffness measurement; kPa, kilopascals; IQR, inter-quartile range; ELF, enhanced liver fibrosis; NFS, non-alcoholic fatty liver disease fibrosis score; ALT, Alanine transaminase; AST, Aspartate aminotransferase.

The most common NIT used was NFS (30%; n = 613), followed by ELF (30%; n = 610), Fib-4 (20%; n = 405), TE (15%; n = 298), and AST:ALT ratio (5%; n = 120).

In the univariable regression analysis (Table 3), MASLD fibrosis was associated with an 88% increased risk of CKD diagnosis relative to MASLD without fibrosis (RR 1.88, CI 1.50–2.36, *p* = <0.0001). In multivariable analysis it remained associated with increased likelihood of CKD, although the estimate attenuated to 31% (*a*RR = 1.31, CI 1.04–1.64, *p* = 0.021) when adjusted for confounders.

**Table 3:** Models of relative risk of chronic kidney disease among those with MASLD fibrosis, both univariable and adjusted for confounders (n = 2,024).

**Variable**	**n (%)** ^†^	**RR (95% CI)**	***p***
MASLD [no fibrosis]	144 (10.1%)		
MASLD fibrosis	112 (18.9%)	1.88 (1.50–2.36)	<0.0001
**Variable**	**n (%)** ^†^	***a*RR (95% CI)**	***p***
MASLD [no fibrosis]	144 (10.1%)		
MASLD fibrosis	112 (18.9%)	1.31 (1.04–1.64)	0.021
Age [≤61]	61 (6.2%)		
>61	192 (19.4%)	2.32 (1.74–3.09)	<0.0001
Sex [female]	111 (12.4%)		
Male	145 (12.8%)	0.94 (0.75–1.18)	0.607
Diabetes [no]	33 (5.7%)		
Yes	223 (15.4%)	2.42 (1.71–3.44)	<0.0001
CVD [no]	149 (9.7%)		
Yes	107 (22.1%)	1.59 (1.26–2.01)	<0.0001
Hypertension [no]	84 (9.1%)		
Yes	172 (15.6%)	1.39 (1.07–1.81)	0.014
BMI [≤30]	119 (14.6%)		
≥31	137 (11.3%)	0.87 (0.70–1.10)	0.241
Nephrotoxic medications, ACEs, ARBs, diuretics [no]	103 (10.3%)		
Yes	153 (14.9%)	1.02 (0.79–1.30)	0.908
Ever smoked [no]	110 (10.6%)		
Yes	146 (14.81%)	1.20 (0.96–1.51)	0.110

**Abbreviations**: RR: risk ratio; *a*RR: adjusted risk ratio; MASLD, metabolic dysfunction associated steatotic liver disease; CVD, cardiovascular disease; BMI, body mass index; ACE, angiotensin-converting-enzyme; ARB, angiotensin receptor blockers.

^†^Sum of cohort at each variable level with chronic kidney disease.

**Fig 3:**
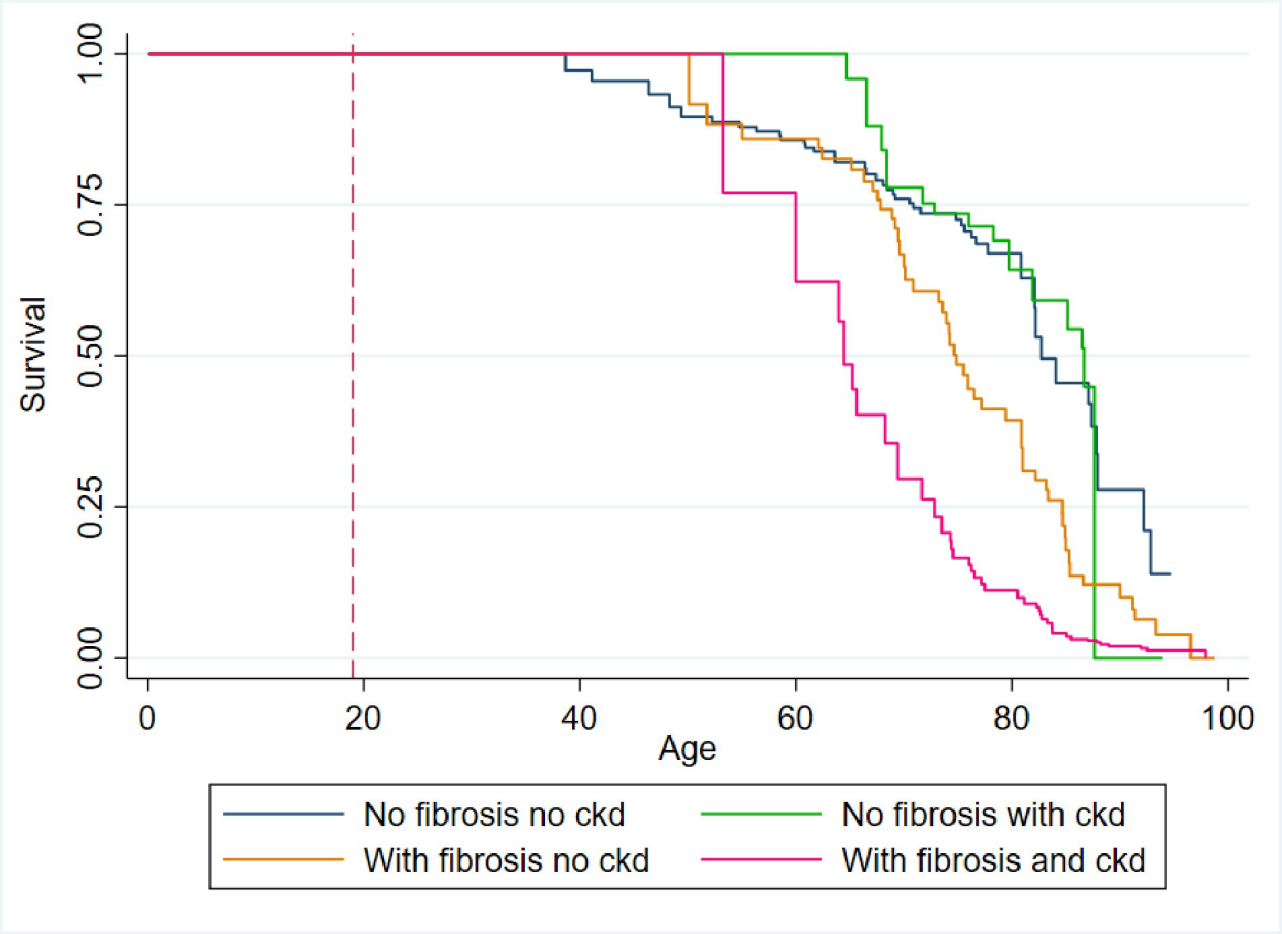
Kaplan-Meier survival curve demonstrating impaired survival according to MASLD fibrosis and CKD status, adjusted for age, sex, cardiovascular disease, and diabetes (n = 2,024). **Note:** Red dotted line on X axis denotes earliest entry age to the analysis (i.e. youngest age at date of screening). This analysis excluded individuals with CKD of structural, autoimmune, or malignant, aetiology; therefore, n = 7 deaths are excluded from the estimates.

Overall, 147 (7.2%) participants died during the observation period. This appeared to vary across MASLD and CKD states. Among those with no fibrosis or CKD, 42 (3.3%) died; among those with no fibrosis but with CKD, 20 (12.4%) died; among those with fibrosis, without CKD, 46 (9.6%) died; while among those with both fibrosis and CKD, 39 (33.3%) died. Seven (4.8%) deaths occurred among participants with CKD of a structural, autoimmune, or malignant, which were excluded from the following survival estimates. Adjusted for age, sex, diabetes, and CVD, the median age of survival among those with no fibrosis or CKD was 82.2 years (IQR 66.5–87.9); among those with no fibrosis, but with CKD, was 71.7 years (IQR 66.5–85.1); among those with fibrosis, without CKD, was 73.9 years (IQR 73.9–81.0); and among those with both fibrosis and CKD was 64.0 years (IQR 53.3–69.4) (Fig 3).

These survival differences were statistically significant (*X*^2^ 49.9, *p* = <0.0001). In line with these estimates, MASLD with fibrosis and CKD was associated with significantly higher hazard of death in both univariable (HR 5.19 [3.20–8.43], *p* = <0.001) and adjusted (*a*HR 5.07 [3.07–8.39], *p* = <0.001) regression analyses, relative to participants with neither fibrosis nor CKD (Table 4).

**Table 4:** Cox models of mortality risk among those with MASLD and CKD, both univariable and adjusted for confounders (n = 2,024).

**Variable**	**n (%)** ^†^	**HR (95% CI)**	***p***
MASLD [no fibrosis, no CKD]	42 (3.3%)		
MASLD no fibrosis, with CKD	15 (10.4%)	1.94 (1.05–3.55)	0.033
MASLD with fibrosis, no CKD	46 (9.6%)	2.36 (1.54–3.63)	<0.001
MASLD with fibrosis and CKD	37 (33.0%)	5.19 (3.20–8.43)	<0.001
**Variable**	**n (%)** ^†^	***a*HR (95% CI)**	***p***
MASLD [no fibrosis, no CKD]	42 (3.3%)		
MASLD no fibrosis, with CKD	15 (10.4%)	1.88 (1.02–3.47)	0.044
MASLD with fibrosis, no CKD	46 (9.6%)	2.30 (1.49–3.56)	<0.001
MASLD with fibrosis and CKD	37 (33.0%)	5.07 (3.07–8.39)	<0.001
Sex [female]	53 (5.9%)		
Male	87 (7.7%)	1.37 (0.97–1.95)	0.074
Diabetes [no]	31 (5.3%)		
Yes	109 (7.6%)	1.09 (0.72–1.66)	0.680
CVD [no]	81 (5.3%)		
Yes	59 (12.2%)	1.04 (0.72–1.50)	0.846

**Abbreviations**: HR: hazard ratio; *a*HR: adjusted hazard ratio; MASLD, metabolic dysfunction associated steatotic liver disease; CVD, cardiovascular disease.

^†^Sum of cohort at each variable level who died.

**Note:** This analysis excluded individuals with CKD of structural, autoimmune, or malignant, aetiology; therefore, n = 7 deaths are excluded from the estimates.

**Univariable model fit:** *X*^2^ 43.78, *p* = <0.0001. Harrell’s C: 0.56 (95% CI 0.50 to 0.62), p <0.0001.

**Adjusted model fit:** *X*^2^ 47.34, *p* = <0.0001. Harrell’s C: 0.58 (95% CI 0.52 to 0.64), p <0.0001.

**Survival information:** n = 2,024; failures = 140, time at risk = 4,619.46 years; earliest entry age = 19.2; last observed exit age = 98.7.

## Discussion

In this study our primary aim was to determine whether liver fibrosis is associated with an increased risk of CKD in a real-world cohort of people living with MASLD. Our analysis implies there is a strong association between hepatic fibrosis and risk of CKD in this cohort, independent of a broad range of important co-occurring risk factors. This suggests that any degree of liver fibrosis, in the context of MASLD, is a potential determinant of renal impairment. Our work builds on studies that have previously implicated a relationship between the two diseases and called for more work in the area to improve our understanding of potential pathophysiological mechanisms at play [10, 19]. It further aligns with previous international work that identified a correlation between severity of fibrosis among people with MASLD and prevalence of renal impairment [32–34]; this effect has also been shown in people with type 1 diabetes and MASLD, where CKD prevalence can be significantly higher amongst those with fibrosis despite adjusting for renal risk factors [35]. Some particular strengths of our study are the utilisation of uACR results for a more comprehensive definition of CKD, the broader range of confounders included in adjusted analyses, and the inclusion of a survival analysis to elucidate mortality implications of people living with these co-morbidities. Our cohort exhibits typical co-occurring metabolic conditions, with a considerable prevalence of obesity, CVD, and hypertension [36–38]. Around two-thirds of participants had diabetes, which is considerably higher than the typical prevalence of 22.5% (17.9–27.9) found in other studies [34]. This may in part be due to the MASLD diagnostic criteria enhancing the detection of diabetes relative to NAFLD, as others have found [39]. One third of our cohort had MASLD with fibrosis, which is in-keeping with the estimated fibrosis progression rate in people living with MASLD (40.8%; 95% CI 34.7–47.1) [37]. These results have important implications for MASLD surveillance programs and will be useful, we believe, to those designing early interventional pathways for people living with MASLD and associated co-morbidities.

Although a link between fibrosis and CKD in MASLD is becoming clearer, the pathophysiological mechanism underpinning this relationship remains uncertain [18]. As a parallel, hepatorenal syndrome (HRS), where liver cirrhosis (of any aetiology) causes renal impairment via increased splanchnic blood flow, and consequent systemic hypovolaemia and renal hypoperfusion, may offer some pathogenic insight, although this is unlikely responsible in those with less advanced fibrosis [40]. The manifestation of this circulatory dysfunction in the earlier stages of MASLD (i.e. fibrosis rather than cirrhosis) is one possible mechanism for MASLD-induced kidney damage. There is evidence of early development of sub-clinical portal hypertension in pre-cirrhotic MASLD, [41] and this could potentially be a driver of CKD in MASLD fibrosis in those with more advanced fibrosis. Alternatively, there is an association between glomerular hyperfiltration—a compensatory increase in eGFR known to be an early indicator of kidney disease [42]—and liver stiffness in those with MASLD [43]. This effect may be mediated through obesity, as central obesity is associated with renal hyperfiltration [44] and is strongly associated with MASLD. These two mechanisms provide plausible explanations for the MASLD fibrosis-CKD association observed in this study, although we were not able to determine whether the MASLD or CKD occurred first in our cohort and whether severity imparted relative bias. Although liver fibrosis was an independent risk factor for CKD in this cohort, given the close synergies between MASLD, MetS, and CKD, an indirect mechanism mediated through diabetes or atherosclerotic diseases is also a plausible explanation which warrants further study.

Irrespective of the underlying mechanisms, the increased risk of CKD amongst our MASLD-fibrosis participants illustrates the multi-system health consequences of worsening fibrosis among people with MASLD. Additionally, the differences we observed in mortality suggest that once people have developed both MASLD fibrosis and CKD they are at significantly heightened mortality risk. A recent study showed those living with co-occurring CKD and MASLD were at a higher risk of cardiovascular events and all-cause mortality than those without MASLD [45]. There is also evidence that MASLD increases the rate of progression to end-stage renal disease amongst those with co-occurring CKD [46, 47]. Increasing liver fibrosis has been shown to lead to higher all-cause mortality, [48] and future studies might consider the compounding impacts of co-occurring diseases such as CKD in the MASLD context, as well as establishing the most common causes of mortality in people with both conditions. Further, our results suggest there is a compounding effect of having both fibrosis and CKD, where the mortality risk of having both conditions exceeds the sum of each. The link between multi-morbidity and mortality has been illustrated in other studies, usually in older populations, but more specific hepatic or renal mechanisms could be at play here, which warrants further study [49]. Identifying people with dual diagnoses of MASLD and CKD is important to combat the rising disease and mortality burden of MASLD, and increasing CKD prevalence, like MASLD, is expected to substantially impact on health service delivery globally in the medium term [50, 51]. While the estimated overall MASLD population is considerable, [52] those with fibrosis are a comparatively smaller cohort, although specific populations are at greater risk; i.e. those with diabetes [53]. As previously noted, the MASLD definition may lead to higher detection of diabetes in affected patients, which implies GFR screening among MASLD patients may be a cost-effective approach to implement within surveillance programmes. Once MASLD fibrosis is established, screening these individuals for CKD may be appropriate. Although studies have demonstrated population-based CKD screening using GFR is cost ineffective, as is screening among sub-groups with hypertension or older people, it has been demonstrated to be cost-effective among other risk groups, such as those with diabetes [54]. Crucially, early identification of CKD allows additional treatments that modify the disease course, [55] and with strong multi-speciality teamworking screening of people with MASLD fibrosis could improve health outcomes.

With an ageing population and rising obesity rates, MASLD prevalence is predicted to increase rapidly. There is consequently an impending global challenge to meet the large-scale health needs of the MASLD population [6, 56]. The estimated healthcare costs associated with MASLD are nearly twice as high as those without MASLD, adjusted for age [57]. It is also clear from the high rates of obesity and comorbidities within this cohort, and other MASLD studies, [9] that this is a multi-system disease and cross-disciplinary collaboration between clinical specialties is important to address MASLD-related morbidity and mortality. To meet this challenge, there is an ongoing necessity for scalable diagnostic systems, to provide population-level screening and targeted interventions to ease the clinical burden on healthcare professionals. Our iLFT system uses automated screening on whole blood samples for multiple aetiologies, which is both effective and cost effective in diagnosing MASLD and is one scalable solution available [21]. To ensure appropriate care and prioritisation of clinical resources, these systems must be guided by evidence-based clinical guidelines. Currently, the American Association for the Study of Liver Disease recommends performing uACR for people with suspected MASLD, [58] and with the evidence from this study and others, [32, 33] the addition of eGFR screening for those with fibrosis appears appropriate. Similarly, current European guidelines on the management of MASLD could consider the addition of renal screening for people with MASLD who have evidence of fibrosis [59].

### Limitations

There are several limitations of this study. First, this study does not give insight into the chronology of MASLD fibrosis and CKD. Determining whether these individuals are more likely to develop fibrosis before or after CKD is important to ascertain the natural history of this fibrosis-CKD relationship. Second, although we adjusted for specific co-medications in the regression analysis we could not determine when people were on or off medications, meaning we may not have accurately estimated the impact and associated risk, which will have varied over time. Third, routinely collected healthcare data is prone to errors and possible biases, including linkage problems and mischaracterisation at input [60]. This was minimised by using unique patient identifiers, allocated nationally across primary and secondary care, as well manual checking of inconsistencies at the individual level. Fourth, we did not exclude diabetes through oral glucose tolerance tests for individuals with borderline HbA1c levels, instead they were considered non-diabetic; we acknowledge this creates some uncertainty. However, we did use a cut-off value for diagnosis which is recommended in UK clinical guidelines [61]. Finally, this work was completed in a regional setting within a health service that is free at the point of need, therefore the international generalisability of the findings is inherently limited.

## Conclusion

Our study demonstrates that, among people living with MASLD, hepatic fibrosis is a critical risk factor for CKD, potentially on a similar order of significance to well-known causes of renal impairment. The physiological basis for this relationship remains uncertain, but future work could investigate subclinical hepatorenal syndrome or obesity-driven renal hyperfiltration as possible causative mechanisms. The results also highlight a need to screen people with MASLD fibrosis for renal impairment. Furthermore, mortality in this group suggests a higher risk for people with MASLD fibrosis and CKD compared to those without, highlighting the crucial need for scalable screening systems to enable intervention early in the disease course. Future work in this area could include studying of the timeline of fibrosis and CKD diagnoses and exploration of pathophysiological mechanisms, and robust cost-effectiveness analyses of renal screening within MASLD care pathways.

## Supporting information

S1 Checklist Checklist of items that should be included in reports of cohort studies. (DOCX)
